# The management of metastatic GIST: current standard and investigational therapeutics

**DOI:** 10.1186/s13045-020-01026-6

**Published:** 2021-01-05

**Authors:** Ciara M. Kelly, Laura Gutierrez Sainz, Ping Chi

**Affiliations:** 1 Department of Medicine, Memorial Sloan Kettering Cancer Center, New York, USA; 2 Department of Medicine, Weill Cornell Medical College, New York, USA; 3 Human Oncology and Pathogenesis Program, Memorial Sloan Kettering Cancer Center, New York, USA; 4 Department of Medical Oncology, Hospital Universitario La Paz, IdiPAZ, Madrid, Spain

**Keywords:** Gastrointestinal stromal tumor, Metastatic GIST, Imatinib, Tyrosine kinase inhibitors

## Abstract

Gastrointestinal stromal tumor (GIST) is the most common mesenchymal tumor of the gastrointestinal tract. The majority of GISTs harbor gain of function mutations in either KIT or PDGFRα. Determination of the GIST molecular subtype upon diagnosis is important because this information informs therapeutic decisions in both the adjuvant and metastatic setting. The management of GIST was revolutionized by the introduction of imatinib, a KIT inhibitor, which has become the standard first line treatment for metastatic GIST. However, despite a clinical benefit rate of 80%, the majority of patients with GIST experience disease progression after 2–3 years of imatinib therapy. Second and third line options include sunitinib and regorafenib, respectively, and yield low response rates and limited clinical benefit. There have been recent FDA approvals for GIST including ripretinib in the fourth-line setting and avapritinib for *PDGFRA* exon 18-mutant GIST. This article aims to review the optimal treatment approach for the management of patients with advanced GIST. It examines the standard treatment options available but also explores the novel treatment approaches in the setting of imatinib refractory GIST.

## Background

Gastrointestinal stromal tumor (GIST) is the most common soft tissue sarcoma subtype. The incidence of GIST is between 10 and 15 cases per million worldwide and ~ 5000 in the USA [1, 2]. The median age at diagnosis is 66–69 years, with equal distribution of men and women [1, 2]. GIST originates from the interstitial cells of cajal (ICCs), and it can arise from any part of the gastrointestinal tract, most commonly in the stomach (55.6%), followed by the small bowel (31.8%), colorectum (6%), other locations (5.5%) and the esophagus (0.7%) [1].

The diagnosis of GIST relies on the combination of the clinical scenario, the tumor’s anatomic location, the immunohistochemistry (IHC) patterns, as well as molecular features. The majority of GIST are immunohistochemically positive for KIT (CD117) and DOG-1 [3]. Other IHC markers frequently expressed in GIST include CD34 antigen (70%), smooth muscle actin (SMA, 30–40%), S100 protein (10%) and desmin (< 5%) [6].

### Molecular classification of GIST

**Fig. 1:**
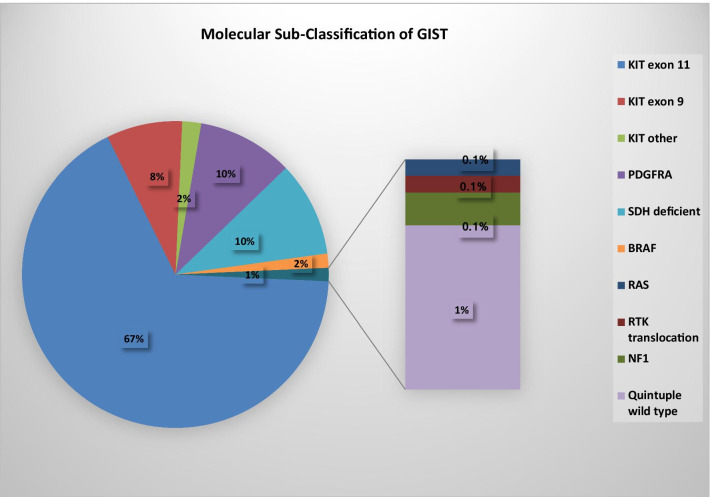
Molecular sub-classification of GIST

The majority of GIST (75–80%) harbor gain of function *KIT* mutations (Fig. 1). Exon 11 of KIT is the most frequently mutated region, affecting approximately two-thirds of GIST. In-frame deletions in KIT exon 11, particularly those involving codons 557 and 558, are associated with a worse prognosis compared to KIT exon 11 point mutations [4]. Mutations in *KIT* exon 9 occur in approximately 8–10% of GIST and are most commonly associated with small or large bowel tumors. Primary mutations in *KIT* exons 13, 17 and 18 are rare. Platelet-derived growth factor receptor alpha (*PDGFRA*)-mutant GIST represents the next most common molecular subtype, occurring in approximately 10% of GIST and generally arises in the stomach [5]. Exon 18 of *PDGFRA* is the most frequently mutated region, affecting approximately 8% of GIST. *PDGFRA* exon 18 *D842V* mutations account for 70% of *PDGFRA* mutant cases [7]. Rarely *PDGFRA* mutations occur within exon 12 or 14 [5].

Approximately 10–15% of GISTs are *KIT* and *PDGFRA* wild-type and are associated with genetic alterations in RAS-MAPK pathway (gain-of-function *RAS/BRAF* mutations or loss of function neurofibromatosis type 1 [*NF1]* mutations) or succinate dehydrogenase (SDHA/B/C/D*)* deficiency*. SDHA/B/C/D* deficiency GIST may be caused by a germline inactivating mutation in the suppressor genes encoding the SDH complex (SDHA, SDHB, SDHC and SDHD subunits) or by SDHC promotor-specific CpG island hypermethylation (SDHC epimutation) [5]. Other new molecular sub-classifications within GIST are oncogenic RTK translocation associated GIST or quadruple wild-type GISTs and quintuple wild-type GIST which refer to GISTs that are devoid of mutations in *KIT/PDGFRA/RAS-MAPK* pathway/*SDH* complex and oncogenic RTK translocations [5–7].

GISTs are generally resistant to conventional chemotherapy. The survival of metastatic GIST has greatly improved since 2002, when the US Food and Drug Administration (FDA) approved imatinib mesylate [8]. *KIT* exon 11 and 9 mutant GISTs are sensitive to imatinib. *KIT* exon 11 mutant GIST yields significantly higher response rates to imatinib and has longer overall survival than those with *KIT* exon 9 mutant or *KIT*/*PDGFRA* wild type GIST [9]. *PDGFRA* exon 18 *D842V* mutant GIST is resistant to imatinib. Avapritinib was recently FDA approved for this indication. Beyond this, a clinical trial should always be considered. Other *PDGFRA* mutant GISTs are generally responsive to imatinib. *KIT* and *PDGFRA* wild-type GIST have no standard/effective therapeutic alternative; therefore, clinical trial options should always be considered [5].

Knowledge of the molecular landscape in GIST is important because it provides prognostic information but also guides therapeutic selection. This review aims to discuss the management of advanced GIST focusing on the standard-of-care therapeutic options and novel therapeutics in clinical investigation.

### Medical management of advanced GIST

#### Imatinib-sensitive GIST: first line therapy

Imatinib is a selective, small molecule inhibitor of three receptor tyrosine kinases: the transmembrane receptor *KIT*, the chimeric *BCR-ABL* fusion oncoprotein of chronic myeloid leukemia and *PDGFRA*. In 2002, the FDA approved imatinib for the management of patients with advanced GIST. This accelerated approval was based on the results of a multi-center, randomized study that evaluated the safety and efficacy of imatinib at two dose levels (either 400 mg and 600 mg daily) in 147 patients with advanced GIST [8]. The clinical benefit rate of imatinib was 81%; with 53.7% of patients achieving a RECIST partial response. The median time to an objective response was 3 months. Almost 14% of patients had early progressive disease despite imatinib therapy. No complete responses were observed. The majority of patients experienced mild to moderate adverse effects. The most common adverse events included edema (74%, frequently periorbital in location), nausea (52%), diarrhea (45%), myalgia or musculoskeletal pain (40%), fatigue (35%), dermatitis or rash (31%), headache (26%) and abdominal pain (26%).

The optimal imatinib dose (400 mg daily) to use in the first line setting was determined in two-phase III trials that randomized patients with advanced GIST to receive imatinib at 400 mg or 800 mg daily. The EORTC 62005 trial enrolled 946 patients. The higher imatinib dose arm yielded a statistically significant progression free survival (PFS) rate of 56% versus 50% for the lower dose arm, (estimated hazard ratio 0.82 [95% CI 0.69–0.98]; *p* = 0.026) [10]. The overall response rates were similar between the two treatment arms. The higher dose arm was associated with greater toxicity. The SWOG S0033/CALGB 15105 trial enrolled 746 patients. After a median follow-up of 4.5 years, no significant difference was observed with respect to median PFS (18 vs. 20 months), median OS (55 vs. 51 months) and objective response rate (40% vs. 42%), between the standard dose and the higher dose arms, respectively [11].

In the EORTC 62005 trial, the strongest prognostic factor of risk for progression and death was the presence of a *KIT* exon 9 mutation. In patients with KIT exon 9 disease, the PFS was significantly longer in patients that received double-dose imatinib compared to those that received the standard dose. For patients that crossed over to double-dose imatinib, the response rates observed were significantly higher among patients with *KIT* exon 9 (57%) compared to *KIT* exon 11 (7%) mutant disease. The phase III SWOG S0033/CALGB 15105 trial failed to demonstrate a PFS benefit favoring the *KIT* exon 9 mutant cohort that received the higher dose imatinib. However, it did show improved response rates in the *KIT* exon 9 mutant cohort compared to the *KIT* exon 11 mutant cohort that crossed over to receive double-dose imatinib (67% vs. 17%, respectively). A preplanned meta-analysis of both the EORTC 62005 and SWOG S0033/CALGB 15105 studies confirmed a PFS advantage in favor of patients with *KIT* exon 9 mutant disease who received double-dose imatinib (400 mg, twice daily) compared to standard dose [12]. As a result, the standard dose for patients with *KIT* exon 9 mutant advanced GIST receiving first-line imatinib is 400 mg twice daily.

#### Imatinib refractory GIST

Sunitinib is another small molecule, receptor TKI. It targets *KIT/PDGFRα,* colony stimulating factor 1 receptor (CSF1R), Fms-related tyrosine kinase 3, *RET* and has anti-angiogenic activity by inhibiting vascular endothelial growth factor receptors (*VEGFR1-3*). An early phase study provided safety and promising efficacy data for sunitinib (50 mg, 4 weeks on, 2 weeks off schedule). This led to a randomized, double-blind, placebo-controlled, phase III trial of sunitinib in patients with advanced, imatinib refractory/intolerant GIST. The objective response rate with sunitinib was 7% (RECIST partial response). However, the study demonstrated a significantly longer time to progression, based on RECIST criteria, of 27.3 weeks versus 6.4 weeks in the placebo arm; *p* < 0.0001) [13]. FDA approved sunitinib in the management of advanced GIST in 2006. Another phase II study examined the benefit of an alternative dosing schedule of sunitinib delivered on a continuous, daily basis (37.5 mg once daily) to patients with advanced, imatinib refractory GIST. The clinical benefit rate was 53%, and median PFS was 34 weeks. Most adverse events encountered were ≤ grade 2 in severity and manageable. Morning or evening dosing was comparable with respect to efficacy and safety [14]. A continuous daily dosing schedule of sunitinib is commonly used to treat patients with advanced GIST.

The GIST molecular genotype has been shown to correlate with efficacy of sunitinib. Higher response rates were observed among GIST patients treated with sunitinib that harbored primary *KIT* exon 9 mutations compared to *KIT* exon 11 mutations (58% vs. 34%, respectively). Among patients with advanced GIST treated with sunitinib alone, longer PFS and OS were seen for those with exon 13 or 14 secondary *KIT* mutations compared to those with exon 17 or 18 secondary *KIT* mutations [15].

Regorafenib is another oral multi-targeted kinase inhibitor that targets a number of pathways involved in tumor growth including tumor angiogenesis (*VEGFR*-1, -2, and -3, and *TIE2*), oncogenesis (*KIT, RET, RAF-1, BRAF and BRAFV600E*), and the tumor microenvironment [PDGFR and fibroblast growth factor receptor (FGFR)]. The safety of regorafenib was first demonstrated in a phase I study that enrolled an unselected population of patients with solid tumors. This study confirmed the recommended phase II dose of 160 mg daily (3 weeks on, 1 week off schedule) [16]. A phase II GIST specific study investigated the efficacy of regorafenib among 34 patients with advanced GIST who had previously progressed on at least imatinib and sunitinib. The clinical benefit rate observed was 79% (4 patients achieved a partial response and 22 patients had stable disease at ≥ 16 weeks). The median PFS was 10 months [17]. Regorafenib was subsequently investigated in a phase III study that randomized 240 patients with advanced GIST to receive regorafenib versus placebo plus best supportive care in a 2:1 fashion. The study met its primary endpoint by demonstrating a significant improvement in median PFS (4.8 months and 0.9 months, respectively (hazard ratio [HR] 0.27, 95% confidence interval [CI] 0.19–0.39; *p* < 0·0001). A best response of partial response or stable disease was observed in 75.9% on regorafenib and 34.8% in the placebo arm. The disease control rate was also higher in the regorafenib arm compared to placebo, (52.6% and 9.1%, respectively) [18]. In 2013, the FDA approved regorafenib for use in the management of patients with advanced GIST refractory to imatinib and sunitinib.

A recent phase Ib trial tested the efficacy of rapid alternation of sunitinib and regorafenib: 3 days of sunitinib followed by 4 days of regorafenib, and repeated. Toxicity was encountered at the previously determined optimal doses of both agents. The recommended phase II dose was identified as sunitinib 37.5 mg and regorafenib 120 mg. Stable disease was the best response observed in 4 out of 14 participants. The median PFS was 1.9 months. Rapid alternation of tyrosine kinase inhibitors (TKIs) was an unprecedented investigational approach in GIST that may prove effective when drugs with complementary pharmacokinetics are combined in an effort to minimize toxicity while allowing optimal drug doses to be used [19].

Ripretinib is a potent switch pocket control inhibitor of KIT and PDGFR kinases and has activity against a broad range of mutations. The phase I study enrolled 142 patients with advanced GIST with *KIT* (95%) or *PDGFRα* (5%) mutations. The recommended phase II dose of ripretinib was 150 mg once daily. The ORR in second-, third- and fourth-line patients was 19.4%, 14.3% and 7.2%, respectively [20]. The INVICTUS, phase III, randomized, double-blind, placebo-controlled trial randomized 154 patients with advanced GIST in a 2:1 fashion to receive ripretinib or placebo. GIST patients had progressed on or were intolerant of imatinib, sunitinib and regorafenib. The study met its primary end point showing a significant improvement in median PFS favoring the ripretinib arm [6.3 months vs. 1·0 months (hazard ratio 0.15, 95% CI 0.09–0.25; *p* < 0.0001)]. The median OS was 15.1 months in the ripretinib group compared to 6·6 months in the placebo group (HR 0.36, 95% CI 0.21–0.62). Ripretinib yielded an objective response rate (ORR) of 9%. There was no response observed in the placebo arm. Ripretinib was generally well tolerated. The most common (≥ 20% of patients in the ripretinib group) treatment-related adverse events were alopecia, myalgia, nausea, fatigue, palmar–plantar erythrodysesthesia and diarrhea [21]. In May 2020, the US FDA approved ripretinib for patients with advanced GIST that had progressed on three or more receptor tyrosine kinase inhibitors including imatinib [22]. Intrigue, a phase III trial examining the efficacy of ripretinib compared to sunitinib in patients with advanced GIST who have progressed on or are intolerant of imatinib in the second line setting is currently ongoing [23].

#### Primary imatinib-resistant GIST/PDGFRA D842V-mutant GIST: first line therapy

*PDGFRA*-mutant GIST accounts for 5–10% of GIST and exhibits primary resistance to imatinib and sunitinib therapy. The median PFS of *PDGFRα D842V* mutant advanced GIST on placebo from retrospective analysis was reported to be 2.8 months [24]. Notably, the median PFS of unselected patients with GIST on placebo is less than 6 weeks [13, 18]. This highlights that *PDGFRA D842V* mutant GIST is clinically distinct from imatinib-sensitive disease. It often follows a more indolent disease course. Until January 2020, there was no standard of care systemic therapy available for this GIST molecular subtype and surgical resection was preferred in the setting of small volume disease progression. In a phase II trial, dasatinib yielded a response in one patient with *PDGFRA D842V*-mutant GIST. Based on this preliminary efficacy, dasatinib was added to NCCN compendium of approved agents for this molecular subtype [25].

Avapritinib is a potent, highly selective oral inhibitor of *PDGFRα* mutant kinases. The Navigator, phase I dose escalation/dose expansion study enrolled 231 patients with advanced GIST: *PDGFRA* exon 18 *D842V* mutant = 56 (24%), non-*D842V* mutant = 8 (4%) and *KIT* mutant disease = 167 (72%). In the *PDGFRA D842V*-mutant population, the ORR was 88%. There were five (9%) complete responses and 44 (79%) partial responses. The clinical benefit rate was 98%, which is remarkable in a GIST molecular subtype that was known to be refractory to all previously FDA-approved TKIs for GIST [26]. Avapritinib was generally tolerated. Cognitive effects emerged as an adverse event of special interest. Most cognitive effects, primarily driven by memory impairment, were grade 1 and generally manageable with dose modification. However, 8.3% of patients discontinued avapritinib for a treatment-related toxicity, of which 2% discontinued treatment because of intracranial bleeding [26]. The recommended phase II dose was determined to be 300 mg once daily and the FDA approved avapritinib in January 2020 for adults with inoperable GIST harboring a *PDGFRA* exon 18 mutations, including the *D842V* mutation.

### Imatinib refractory GIST: alternative options

#### Enrollment in a clinical trial

Resistance to imatinib appears to confer a general resistance to non-selective TKIs. A number of novel therapeutic approaches to treat GIST are being investigated.

#### Next-generation selective tyrosine kinase inhibitors

Secondary *KIT* mutations are found in 50–67% of patients with secondary imatinib resistant, *KIT* mutant disease [27, 28]. They commonly arise in *KIT* exons 17, 13 and are less frequently observed in exons 18, 14 or within the *PDGFRA* gene. Next-generation TKIs targeting specific secondary *KIT* mutations are under investigation. Examples of selective TKIs include PLX3397, PLX9486 and AZD3229.

In preclinical models, PLX3397 appears to be a more potent *KIT* inhibitor than imatinib [29]. PLX9486 has selective activity against primary *KIT* mutations (exons 9 and 11) and activation loop mutations (exons 17 and 18). A phase I study of PLX9486 in GIST examined safety up to a dose of 1000 mg daily. The clinical benefit rate was 64%, and the median PFS was 6 months. Analysis of circulating tumor deoxyribonucleic acid (ctDNA) in this cohort confirmed the selectivity profile of PLX9486 with reductions in *KIT* ex 11, 17 and 18. PLX9486 (500 mg qd) was examined in combination with PLX3397 (600 mg qd). The median PFS for this combination was 6 months. Reduction in ctDNA levels of *KIT* exons 13 and 14 was not observed [30]. PLX9486 was also explored in combination with sunitinib creating synergistic potential against a broader range of KIT mutations by blocking active and inactive conformations. A total of 18 patients received this combination therapy. This was a heavily pre-treated cohort, 67% had received three or more prior lines of therapy. The maximum tolerated dose was not reached. The recommended phase II dose was PLX9486 1000 mg daily and sunitinib 37.5 mg once daily administered in 28 day cycles. The safety profile of the combination therapy was comparable to that of single agent sunitinib. The ORR was 20%. The median duration of response was 10 months. The median PFS for the combination therapy was 12 months [31, 32].

In preclinical models, avapritinib was highly active against imatinib-resistant patient derived GIST xenografts. The “Navigator,” phase I study of avapritinib demonstrated promising activity in ≥ 4th line patients (*n* = 109), 3rd line [regorafenib-naïve] (*n* = 23) and 2nd line *non-D842V* mutant GIST (*n* = 20) the ORR was 20%, 26% and 25%, respectively. The median duration of response was 7–10 months in the ≥ 4th line and 3rd line cohorts. Best response by mutational profile in the ≥ 4th line cohort failed to demonstrate activity in tumors harboring *KIT V654A* and *T6701* mutations [33]. The “Voyager,” randomized, phase III study of avapritinib compared with regorafenib in 3rd or 4th line *KIT/PDGRFA* mutant GIST enrolled 476 patients [34]. In April 2020, a press release regarding the Voyager trial reported no statistically significant difference in median PFS between the avapritinib arm (4.2 months) and the regorafenib arm (5.6 months). The ORR was 17.1% in the avapritinib arm and 7.2% in the regorafenib arm.

AZD3229 is an oral, potent, pan-KIT/PDGFRα inhibitor. It has demonstrated activity in-vivo and in-vitro preclinical, GIST models across a broad range of primary and secondary *KIT/PDGFRA* mutations that confer resistance to standard of care agents [35]. This compound was designed to reduce activity against VEGFR-2 thereby, attempting to minimize off-target effects which often leads to sub-optimal dosing with standard of care agents used in GIST [36]. This agent demonstrates promising pre-clinical activity in GIST tumor models and merits investigation in the clinical setting.

#### Targeting ETV1

ETS variant transcription factor 1 *(ETV1)* is a lineage specific transcription survival factor that is highly expressed by GIST cells and their precursor cells the interstitial cells of cajal. *ETV1* promotes the growth and survival of both the ICC and GIST. *KIT* mutant GIST results in constitutive activation of the *MAP* kinase pathway. This stabilizes the *ETV1* transcriptional output, which promotes GIST tumorigenesis. *ETV1* may also be critical for the survival and maintenance of the intrinsically imatinib-resistant, *KIT*-independent stem cell/progenitors in GIST, suggesting that this may be a therapeutic target to consider in *KIT* wild-type GIST [32]. Combination inhibition of *MAP* kinase and *KIT* signaling represents a promising therapeutic approach. In preclinical studies, the combination of imatinib and binimetinib destabilized the *ETV1* protein resulting in greater suppression of GIST cell growth compared to either drug alone and also induced apoptosis [37].

The combination of imatinib and binimetinib was investigated in patients with advanced GIST of any genotype. The phase Ib/II trial demonstrated safety and identified the recommended phase II dose (imatinib 400 mg daily and binimetinib 30 mg twice daily). The phase II study met its primary endpoint by demonstrating an ORR of 66.7% (*n* = 26/39) in the first-line management of advanced GIST. The median duration of response was 30 months. Notably, the rate of conversion to resection was 88.9% (95% CI, 52–100%)] among 9 patients who had initially inoperable disease. The median PFS for the combination therapy was 29.9 months, and the median overall survival had not yet been reached [38]. These results compare very favorably with imatinib alone and warrant further evaluation in a larger, randomized, controlled, phase III trial (Table 1).

**Table 1:** FDA approved systemic therapies for the management of advanced GIST

Drug	*N*	Number of prior therapies (median)	Phase	Randomized	Other arm	RR	CBR	PFS (median)	OS (median)	Year	References
Imatinib (first-line)	147	Any	II	Yes	400 mg versus 600 mg daily	53.7% (overall)	81.6% (overall)	–	–	2002	[8]
	946	Any	III	Yes	400 mg daily versus 400 mg twice daily	52% (overall)	84% (overall)	–	–	2004	[10]
	746	Any	III	Yes	400 mg daily versus 800 mg daily	45%	67–70%	18 mo (400 mg daily) versus 20 mo (800 mg daily)	55 mo (400 mg daily) versus 51 mo (800 mg daily)	2008	[11]
Sunitinib (second-line; 50 mg daily × 28 days, 14 days break)	312	1	III	Yes (2:1 in favor of sunitinib arm)	Placebo	7% (sunitinib) versus 0% (placebo)	65% (sunitinib) versus 37% (placebo)	27.3 wks (sunitinib) versus 6.4 wks (placebo)	–	2006	[13]
Sunitinib (second-line; 37.5 mg daily)	60	1	II	No	–	13%	53%	34 weeks	107 weeks	2009	[14]
Regorafenib (R) (third-line)	199	2	III	Yes (2:1 in favor of R arm)	Placebo	4.5% (R) versus 1.5% (placebo)	75.9% (R) versus 34.8% (placebo)	4.8 mo (R) versus 0.9 mo	–	2013	[18]
Ripretinib (fourth-line)	111	At least 3 (imatinib, sunitinib and regorafenib)	III	Yes (2:1 favoring ripretinib)	Placebo	9% (ripretinib) versus 0% (placebo)	75% (ripretinib) versus 20% (placebo)	6.3 mo (ripretinib) versus 1.0 mo (placebo)	–	2020	[21]
Avapritinib (*PDGFRA* D842V mutant − first line +)	56	Any	I	No	–	88%	98%	N/A	N/A	2020	[26]

N, number of patients; RR, response rate; CBR, clinical benefit rate; wks, weeks; PFS, progression free survival; mo, month; vs, versus; N/A, not applicable; OS, overall survival; yr, year published; ref, reference; R, Regorafenib

#### Immunotherapy

The role of the immune system has been explored in preclinical *KIT* mutant GIST mouse models. Imatinib treatment induces a dramatic increase in CD8 + T cell number and proliferation and produces an increased ratio of CD8 + effector T cell: CD4 + regulatory T cells. Imatinib-sensitive *KIT* mutant tumors contained greater frequencies of CD3 + and CD8 + T cells, but a lower percentage of CD4 + T cells and T regs compared to imatinib-resistant KIT mutant GIST tumor mouse models. The anti-tumor effects of imatinib are reduced in the setting of depleted CD8 + but not CD4 + T cells, natural killer (NK) cells or myeloid cells. This underlines the contribution of CD8 + effector T cells to the anti-tumor effect of imatinib. Imatinib has been shown to regulate the intratumoral T cells and immune microenvironment through inhibition Indolamine 2,3-dioxygenase 1 (*IDO1*) [39]. Given the role of CD8 + effector T cells in imatinib response, concurrent administration of imatinib and an immune checkpoint inhibitor (ipilimumab) were tested in KIT mutant GIST mouse models. The combination was shown to be synergistic and significantly reduced tumor size compared to either treatment alone [39].

This preclinical work led to a phase Ib study that examined the combination of dasatinib, a multi-TKI and ipilimumab, an anti-CTLA4 antibody, in patients with advanced sarcomas. Twenty patients had advanced GIST. The combination was shown to be safe, however, not synergistic in its effect. Many patients progressed prior to the first disease assessment at 3 months (median PFS was 2.8 months). The ORR was 0%. The study population was heavily pre-treated (median number of prior therapies = 3) [40]. Dasatinib monotherapy was previously shown to have limited benefit in this setting producing a median PFS of less than 2 months [41]. Preclinical studies previously discussed highlighted that imatinib’s immunostimulatory effect on the tumor immune microenvironment is greatest in the setting of imatinib-sensitive disease. Hence, dasatinib was recognized as a poor TKI choice for the first immunotherapy-based trial in GIST. Combination therapy with an ICI and an approved TKI in a TKI sensitive population warrants exploration.

Two-phase II trials have examined nivolumab with or without ipilimumab in advanced GIST. The first study enrolled 29 patients and demonstrated a clinical benefit rate of 50% (*n* = 8/16) in the nivolumab alone arm compared to 23% (*n* = 3/13) in the combination arm. A partial response was observed in the combination arm. The median PFS was 12.1 weeks in the nivolumab alone arm and 8.3 weeks in the doublet checkpoint inhibition arm [42]. The Alliance A091401 included an expansion cohort in GIST. This cohort demonstrated poor activity for immune checkpoint inhibition among 21 heavily pre-treated patients. The clinical benefit rate was ~ 10% in each arm. No objective responses were seen in either arm. The median PFS was 2.9 months in the doublet arm versus 1.5 month in the monotherapy arm [43].

#### Targeting actionable genomic alterations in GIST beyond known oncogenic drivers

Oncogenic activation of the Phosphoinositide 3-kinase/protein kinase B/mammalian target of rapamycin (PI3K/AKT/mTOR*)* pathway represents a downstream effector in the *KIT* signaling pathway. In preclinical GIST cell line studies, cell growth arrest resulted from PI3K inhibition, and to a lesser degree from MEK/MAPK and mTOR inhibition. In imatinib-resistant GIST cell lines, only PI3K inhibition was shown to halt GIST cell growth and produce apoptosis. Hence, this work postulated that targeting critical downstream signaling proteins in imatinib-resistant disease, such as PI3K, represented a promising therapeutic avenue to explore [44].^`^ Knowledge of the genomic landscape of GIST continues to evolve. Next-generation sequencing is increasingly being utilized in cancer patients in an effort to identify genomic alterations that may represent potential therapeutic targets [45, 46]. Studies examining the molecular spectrum of GIST have identified actionable alterations in the PI3K kinase and mTO*R* pathways, albeit at relatively low frequencies [47].

A phase II study investigated the efficacy of the combination imatinib (600 mg daily)/everolimus (2.5 mg daily) in patients with advanced GIST. A total of 75 patients were enrolled into one of 2 cohorts stratified by line of treatment [36]. There was a high proportion of grade 3/4 adverse events observed in both cohorts [54–75%]. The ORR for this combination was low at 0% and 2% in cohort 1 and 2, respectively. The median PFS was 1.9 months and 3.5 months in cohort 1 and 2, respectively [48]. Early studies using mTOR inhibitors have shown limited success, which may be due to the activation of AKT that occurs following mammalian target of rapamycin complex 1 (mTORC1) inhibition. Therefore, targeting PI3K or AKT*,* which lie upstream of mTORC1, may translate into more complete pathway inhibition.

GIST xenograft preclinical studies have demonstrated enhanced activity for combination of a *PI3K* inhibitors with imatinib compared to either drug alone [49, 50]. A phase Ib trial of Imatinib and an oral PI3K inhibitor, buparlisib enrolled 60 patients with advanced GIST refractory to imatinib and sunitinib. The combination failed to generate an objective response and was not developed further [51].

The most frequently observed gene specific copy number alteration in GIST is within the cyclin-dependent kinase inhibitor 2A *(CDKN2A)* locus that encompasses the tumor suppressors p16 and p14 [47, 52]. French investigators evaluated the efficacy of palbociclib, a CDK4/6 inhibitor, in 71 patients with advanced imatinib- and sunitinib-refractory GIST, 29 had confirmed *CDKN2A* gene loss. An interim analysis revealed that 86% of evaluable patients experienced progression of disease at 4 months. *CDKN2A* status was not shown to correlate with survival or outcome to prior therapy [53].

### Absence of an appropriate clinical trial

In the absence of an appropriate clinical trial, other therapeutic options include (1) an alternative TKI (off-label use), (2) re-challenge with imatinib or other TKI (3) the addition of an mTOR inhibitor to imatinib or sorafenib.

#### Alternative TKIs previously investigated in patients with advanced GIST

A number of other multi-tyrosine kinase inhibitors have been examined in the setting of imatinib refractory advanced disease. These studies were small phase II trials. The drugs investigated include sorafenib, pazopanib, cabozantinib, nilotinib, dasatinib, masitinib, linsitinib, dovitinib, vatalanib and ponatinib [34, 41, 54–63]. The response and survival outcomes reported in these studies are conveyed in Table 2. Some of these drugs are National Comprehensive Cancer Network (NCCN) compendium listed for the management of imatinib refractory advanced GIST. In the absence of a clinical trial, it is reasonable to consider one of these agents where available.

**Table 2:** Alternative TKIs investigated in patients with advanced imatinib refractory GIST

Drug	*N*	Number of prior therapies (median)	Phase	Randomized	Other arm	RR	CBR	PFS (median)	OS (median)	Year	References
Pazopanib^a^	25	2 (at least)	II	No	–	SD-48%	NPR-17%	1.9 mo	10.7 mo	2014	[54]
Pazopanib^a^	81	2	II	Yes	BSC	SD Pazopanib-84%	84%	3.4 versus 2.3 (*p* = 0.03)	17.8 versus 12.9 mo	2016	[55]
						BSC-71%					
Cabozantinib^a^	50	2	II	No	–	PR-14%	80%	6.0 mo	14.4 mo	2019	[56]
						SD-66%					
Sorafenib^a^	31	2	II	No	–	PR-13%	36% at 24 wks	4.9 mo	9.7 mo	2012	[57]
						SD-52%					
Sorafenib^a^	38	2	II	No	–	PR-13%	68%	5.2 mo	11.6 mo	2011	[58]
						SD-55%					
Nilotinib^a^	35	2	II	No	–	PR-3%	29% at 24 wks	3.7 mo	10 mo	2011	[59]
						SD-26%					
Dasatinib^a^	47	2	II	No	–	PR-32%	56%	1.8 mo	19 mo	2011	[41]
						SD-24%					
Ponatinib	45	3	II	No	–	PR-7%	37%	2.0 mo	13.5 mo	2014	[60]
Masitinib	44	1	II	Yes	Sunitinib	–	–	3.7 versus 1.9 mo	29.8 versus 17.4 mo	2014	[61]
Vatalanib	45	1	II	No	–	PR-4.4%	40%	4.5 mo (mTTP)	–	2011	[62]
						SD-35.6%					
Dovitinib	30	2	II	No	–	PR-3%	13%	3.6 mo	9.7 mo	2014	[63]
						SD 10%					
Avapritinib^a^	476	2 or 3	III	Yes	Regorafenib	PR—17.1%(avapritinib) versus 7.2% (regorafenib)		4.2 mo (A) versus 5.6 mo (R)	–	2020	[34]

wt, wild type; N, number of patients; BSC, best supportive care; RR, response rate; PR, partial response; SD, stable disease; CBR, clinical benefit rate; wks, weeks; PFS, progression free survival; mo, month; vs, versus; mTTP, median time to progression; n/a, not applicable; OS, overall survival; yr, year published; ref, reference; A, Avapritinib; R, Regorafenib

^a^NCCN compendium listed

#### Re-challenge with Imatinib or other TKI

In the setting of limited progression, when standard and investigational therapies fail to control disease, consideration can be made to re-challenge with a TKI that was previously tolerated and effective. A retrospective analysis of 71 patients with advanced GIST refractory to imatinib, sunitinib and regorafenib, re-challenged with imatinib (400 mg daily) demonstrated a median time to progression of 5.4 months and overall survival of 10.6 months [64].

Notably, in the setting of progression on TKI therapy, discontinuation of therapy may result in accelerated tumor growth due to withdrawal of treatment for sensitive clones of the disease. Therefore, in the absence of a standard, investigational or clinical trial option, TKI therapy should be continued as an element of best supportive care [2].

#### Addition of an mTOR inhibitor to imatinib or other TKI.

As previously highlighted activation of the mTOR pathway is a downstream effector of KIT signaling. Hence, in the setting of imatinib refractory disease in the absence of standard or clinical trial options the addition of sirolimus to a TKI can be considered. Safety has been confirmed for the addition of sirolimus to sorafenib or sunitinib in patients with advanced cancer [65]. However, we do not have efficacy data for this treatment approach.

#### *PDGFRA D842V*-mutant GIST: investigational options

Crenolanib is a selective inhibitor of PDGFRα and FLT3 with nanomolar activity against *PDGFRα D842V* mutant GIST. A phase I study confirmed an objective response rate of 12.5%  (2/16) and a clinical benefit rate of 31% (partial response, *n* = 2, stable disease, *n* = 3) in heavily pretreated patients with *PDGFRα D842V* mutant GIST. An international, randomized phase III trial of crenolanib versus placebo in 120 patients with *PDGFRα D842V* mutant GIST continues [66].

### Biomarker development in GIST

Molecular classification of GIST is imperative to inform optimal therapeutic selection in the management of GIST. Testing GIST tumor tissue obtained from a surgical or biopsy procedure is the standard diagnostic approach used to determine the molecular landscape of GIST. Intra-tumoral heterogeneity within GIST limits the ability to detect the complete genomic spectrum from testing isolated tumor tissue samples. As previously outlined, the molecular spectrum of GIST may change following exposure to tyrosine kinase inhibition. Many novel therapies under investigation are selective for specific *KIT/PDGFRα* mutations. Hence, there is an emergent need to develop a noninvasive approach to molecularly characterize GIST in a serial manner. CtDNA extracted from plasma may overcome the limitations of tumor tissue. Sequenced ctDNA has detected primary and secondary mutations in patients with advanced GIST [67, 68]. The detection of ctDNA has been shown to correlate with tumor burden and response to treatment in GIST and other tumor types [69–71].

To date, studies examining sequenced ctDNA in GIST have been small and used older sequencing technologies. Sequenced ctDNA represents a candidate blood biomarker of GIST molecular behavior and treatment response that warrants further investigation and validation in a prospective manner. Several clinical trials are incorporating sequencing ctDNA material in an effort to add to this evolving area of research.

An exploratory analysis of ctDNA extracted from patients with *PDGFRA D842V*-mutant GIST enrolled in the phase 1 study of avapritinib was presented at the European Society of Medical Oncology annual meeting in 2018. They found that baseline ctDNA levels were the strongest independent baseline predictive indicator of PFS over established predictive indicators including Eastern Cooperative Oncology Group (ECOG) performance status, lesion size and age. Moreover, in the majority of patients with *PDGFRα D842V-*mutant GIST treated with avapritinib, ctDNA levels fell below the limit of detection (0.05%) by two months on treatment and large declines in on-treatment ctDNA levels were associated with high baseline ctDNA, an independent risk factor for progression. These data indicate that baseline ctDNA levels may have utility as a predictive biomarker in patients with advanced GIST; however, further research is warranted to understand the role and utility of sequenced ctDNA in the management of advanced GIST [72].

## Conclusion and future directions

The management of GIST was revolutionized in 2001 following the introduction of imatinib. Since then the landscape of advanced *KIT*- and *PDGFRA*-mutant GIST management has evolved to include second-, third- and fourth-line TKIs, sunitinib, regorafenib and ripretinib, as well as avapritinib for advanced *PDGFRA D842V* mutant GIST. The obvious clinical challenges that we face now are how to treat patients who have progressed on ripretinib fourth-line therapy, and how to leverage molecular data to optimize the treatment paradigm.

While there has been tremendous progress in the clinical management of advanced GIST, some fundamental challenges remain: (1) imatinib resistance. For the majority (90%+) of patients, they derive clinical benefit to front-line imatinib therapy, but this is not indefinite. Once their disease develops resistance to imatinib, subsequent lines of TKI therapies, including newer generations of TKIs, have limited clinical benefit. Hence, the next phase of clinical investigations should focus on intervening early and developing therapeutic strategies to transform TKI-mediated cytostatic effects to cytotoxic effects. Some of these strategies will involve novel combination therapeutic strategies of targeting different survival pathways for synergistic cell killing effects, and novel therapeutic targets, such as the tumor microenvironment including immune and non-immune stroma and vasculature compartments.

Furthermore, there are significant unmet needs for the subset of patients with *KIT/PDGFRA*-wild type GISTs. These include SDH-deficient GIST mainly affecting pediatric and young adult population, *NF1*-mutant GIST both sporadic and syndromic (associated with type I neurofibromatosis), *RAS*-mutant, *FGFR* mutant GISTs and others. These groups of patients shall be considered differently; and therapeutics development shall be tailored to their particular genetic defects and potential molecular vulnerabilities.

Lastly, a multidisciplinary approach to the management of GIST within a dedicated sarcoma center is the ideal. The addition of surgical resection, radiation therapy and local radiological interventional options to TKI therapy may be appropriate in certain situations but requires careful selection. Clinical trials exploring novel investigational therapies should always been considered.

## Data Availability

Not applicable.

## Abbreviations

GIST: Gastrointestinal stromal tumors
ICCs: Interstitial cells of cajal
IHC: Immunohistochemistry
CD117: Cluster of differentiation 117
DOG-1: Discovered on GIST-1
PDGFRA: Platelet-derived growth factor receptor alpha
NF1: Neurofibromatosis type 1
SDH: Succinate dehydrogenase
RTK: Receptor tyrosine kinase
FDA: Food and Drug Administration
EORTC: European Organization for Research and Treatment of Cancer
SWOG: Southwest Oncology Group
CALGB: Cancer and Leukemia Group B
PFS: Progression free survival
CSF1R: Colony stimulating factor 1 receptor
RECIST: Response evaluation criteria in solid tumors
TKIs: Tyrosine kinase inhibitors
ORR: Objective response rate
ctDNA: Circulating tumor deoxyribonucleic acid
ETV1: ETS variant transcription factor 1
NK: Natural killer
IDO1: Indolamine 2:3-dioxygenase 1
CTLA4: Cytotoxic T-lymphocyte-associated protein 4
PI3K: Phosphoinositide 3-kinase
AKT: Protein kinase B
mTOR: Mammalian target of rapamycin
mTORC1: Mammalian target of rapamycin complex 1
CDKN2A: Cyclin-dependent kinase inhibitor 2A
*NCCN*: National Comprehensive Cancer Network
ECOG: Eastern Cooperative Oncology Group
